# Maintenance chemotherapy in children with ALL exerts metronomic-like thrombospondin-1 associated anti-endothelial effect

**DOI:** 10.18632/oncotarget.3984

**Published:** 2015-05-04

**Authors:** Nicolas Andre, Sylvie Cointe, Vincent Barlogis, Laurent Arnaud, Romaric Lacroix, Eddy Pasquier, Françoise Dignat-George, Gérard Michel, Florence Sabatier

**Affiliations:** ^1^ Service d'Hématologie et Oncologie Pédiatrique, Centre Hospitalo-Universitaire Timone Enfants, AP-HM, Marseille, France; ^2^ Aix-Marseille Université, INSERM, CRO2 UMRS-911, Marseille, France; ^3^ Metronomics Global Health Initiative, Marseille, France; ^4^ Aix-Marseille Université INSERM, Vascular Research Center of Marseille UMRS-1076, Marseille, France; ^5^ Laboratoire d'Hématologie, Centre Hospitalo-Universitaire Conception, AP-HM, Marseille, France

**Keywords:** leukemia, maintenance therapy, metronomic chemotherapy, immune system, angiogenesis

## Abstract

Maintenance chemotherapy is an important part of the treatment of ALL in children. It relies on the long-term oral administration of daily low-dose mercaptopurin and weekly low-dose methotrexate. Although it has been used in the clinic for decades, its mechanisms of action remain unclear. Here, we investigated different angiogenic and immune biomarkers to gain insights into the mechanisms of action of maintenance therapy in children with ALL. We thus monitored circulating endothelial cells (CEC), endothelial progenitor cells (EPC) and endothelial microparticles (EMP), pro-angiogenic factors (VEGF, VEGFR-1 and Ang-2), anti-angiogenic factor thrombospondin-1 (THBS1) and regulatory T lymphocytes (Treg) in 47 children with ALL during the maintenance phase of their treatment (at treatment initiation and after 6, 12 and 18 months). We observed a statistically significant decrease in EPC and EMP counts throughout the maintenance phase associated with a significant increase in THBS1 levels. No significant change was detected in other angiogenic markers or in Treg numbers.

The results presented here indicate that maintenance therapy in children with ALL exerts its antitumor activity at least in part through anti-angiogenic effects, similar to those induced by metronomic chemotherapy. Larger studies are now warranted to validate these findings and determine their clinical implications.

## INTRODUCTION

Maintenance chemotherapy is an important part of the treatment of acute lymphoid leukemia (ALL) in children to prevent relapse by eliminating minimal residual disease [1, 2]. The cornerstone of ALL maintenance therapy relies on the oral administration of daily low-dose mercaptopurin and weekly low-dose methotrexate for a period of time ranging from 2 to 3 years [1]. Interestingly, metronomic chemotherapy (MC), which relies on the frequent administration of chemotherapy at low doses, significantly below the single maximal tolerated dose and with no prolonged drug-free breaks [3, 4], has recently emerged as an alternative strategy to conventional high-dose chemotherapy. Despite accumulating clinical evidence in a variety of human cancers [4], the efficacy of MC is yet to be validated in large randomized phase III trials. However, as originally theorized by Barton Kamen, maintenance therapy in leukemia can be considered, in retrospect, as the early prototype of successful MC [5].

Angiogenesis plays a role in the pathogenesis and progression of hematological malignancies [6] including leukemia and MC has initially been demonstrated to be an antiangiogenic therapy [3]. Tumor angiogenesis can be easily and non-invasively monitored by analysis of circulating endothelial cells (CEC), endothelial progenitor cells (EPC) and endothelial microparticles (EMP), which are established biomarkers of endothelial integrity in peripheral blood. However, new mechanisms of action of MC have been unveiled, such as i) stimulation of anticancer immune response by depletion of regulatory T lymphocytes (Treg) or activation of dendritic cells, ii) re-induction of tumor dormancy, and iii) potential direct effects against cancer stem cells [4]. Thus, maintenance therapy in leukemia, whose mechanisms of action remain unclear [1, 2], could exert metronomic-like effects and represent a form of multi-targeted therapy.

The aim of this study was to monitor the impact of a maintenance treatment on endothelial biomarkers, angiogenic cytokines, and Treg in 47 children with ALL.

## RESULTS

**Figure 1:**
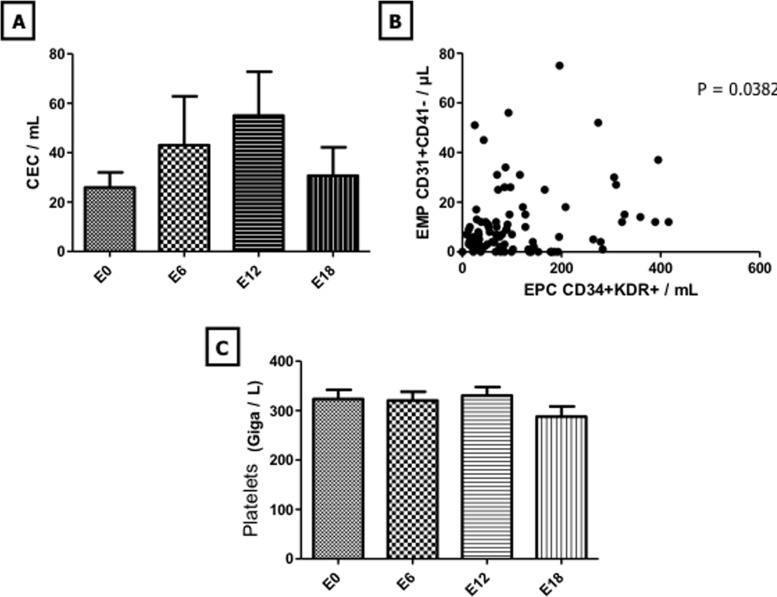
Evolution of CEC and platelet levels during maintenance therapy Evolution of CEC and platelets was monitored at treatment initiation and after 6, 12 and 18 months of maintenance therapy and is shown in panels **A.** and **C.** Correlation between EMP and EPC during the maintenance phase of treatment is shown in panel **B.** *Columns*, mean value from 47 patients; *Bars*, SEM. Abbreviations used: CEC: circulating endothelial cells, EMP: endothelial microparticles, EPC: endothelial progenitor cells.

**Figure 2:**
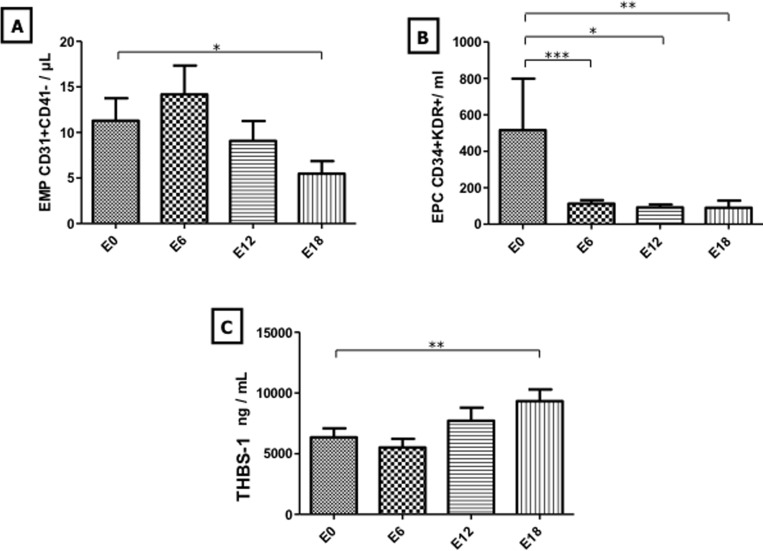
Evolution of EMP, EPC, and THBS-1 levels during maintenance therapy Evolution of EMP, EPC and THBS-1 was monitored at treatment initiation and after 6, 12 and 18 months of maintenance therapy and is shown in panels **A.**, **B.**, and **C.**, respectively. *Columns*, mean value from 47 patients; *Bars*, SEM. Statistical analysis was performed using Wilcoxon matched paired test; *p < 0.05; **p < 0.01; *p* < 0.001. Abbreviations used: EMP: endothelial microparticles, EPC: endothelial progenitor cells, THBS-1: Thrombospodin-1.

**Figure 3:**
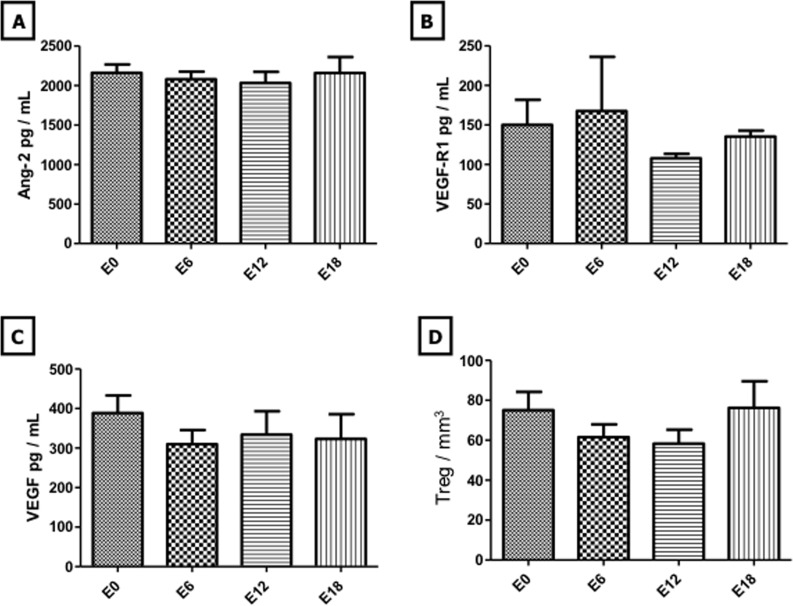
Evolution of angiogenic cytokines and regulatory T lymphocytes during maintenance therapy Evolution of Ang-2, VEGFR-1, VEGF and Treg levels was monitored at treatment initiation and after 6, 12 and 18 months of maintenance therapy and is shown in panels **A.**, **B.**, **C.** and **D.**, respectively. *Columns*, mean value from 47 patients; *Bars*, SEM. Ang-2: Angiopoietin-2, VEGFR-1: Vascular Endothelial Growth Factor Receptor-1, VEGF: Vascular Endothelial Growth Factor, Treg: Regulatory T Lympocyte.

In the 47 children enrolled in this study, endothelial biomarkers analysis indicated no statistically significant change in CEC and platelet counts during maintenance therapy (Figure 1). In contrast, EMP counts decreased at E12 and E18 with a significant change after 18 months of maintenance therapy compared to baseline counts (11.3 +/− 12.84 EMP/μL *vs* 5.47 +/− 1.39 ; *p* < 0.05) (Figure 2A). In line with EMP levels, EPC counts decreased throughout the maintenance phase with values dropping from 507 +/− 272 EPC/mL at E0 to 112.9 +/− 126.6 (*p* < 0.001), 91.06 +/− 83.97 (*p* < 0.05) and 92.32 +/− 181.1 (*p* < 0.01) at E6, E12 and E18, respectively (Figure 2B). Moreover, all of EMP values measured during maintenance treatment correlated with those of EPC (*p* < 0.05) (Figure 1C). Interestingly, THBS-1, which is a well-established mediator of the antiangiogenic activity of MC [7], increased at E12 and E18 with a significant change at E18 compared to baseline (6341 +/− 4121 ng/mL *vs* 9319 +/− 4095; *p* < 0.01) (Figure 2C). In sharp contrast, analysis of other angiogenic cytokines indicated no statistically significant change in VEGF, VEGF-R1 and ANG-2 levels throughout the maintenance phase (Figure 3). There were no significant correlations between biomarkers.

Only one patient (n°14-Table 1) relapsed during the maintenance phase. Noteworthily, this patient displayed high levels of EPC (3092/ml) shortly before relapsing.

The role of the immune system is gaining a considerable interest in solid tumors [8] and is a well-established actor of leukemia treatment. More specifically, Tregs are being increasingly documented as key players in the antitumor activity of MC [4]. However, in this study, Treg counts did not significantly vary during maintenance therapy (Figure 3D).

**Table 1:** Patients characteristics

patient number	age	phenotype (months)	CNS Involvement	hyperleucytosis	Karyotype	FISH	Corticosensitivity	Chemosensitivity
1	154	LAL T	no	yes	abnormal	tel-aml1	yes	yes
2	82	LAL B	no	no	abnormal	negative	yes	yes
3	114	LAL B	no	no	abnormal	negative	yes	yes
4	52	LAL B	no	no	abnormal	negative	no	yes
5	35	LAL B	no	no	abnormal	negative	yes	yes
6	53	LAL B	no	no	abnormal	negative	not evaluable	yes
7	107	LAL B	no	no	abnormal	negative	yes	yes
8	45	LAL B	no	no	normal	negative	no	yes
9	190	LAL B	no	no	abnormal	tel-aml1	yes	yes
10	65	LAL B	no	no	normal	negative	not	yes
11	91	LAL B	no	no	abnormal	negative	ye	yes
12	196	LAL B	no	no	normal	tel-aml1	yes	yes
13	35	LAL B	no	no	abnormal	not done	no	yes
14	180	LAL T	no	no	Failure	negative	yes	yes
15	76	LAL B	no	no	normal	negative	yes	yes
16	61	LAL B	no	no	abnormal	negative	yes	yes
17	183	LAL B	no	no	abnormal	negative	yes	yes
18	91	LAL B	no	no	normal	negative	yes	yes
19	38	LAL B	no	yes	abnormal	negative	yes	yes
20	50	LAL B	no	yes	normal	negative	yes	yes
21	195	LAL B	no	no	normal	tel-aml1	yes	yes
22	78	LAL B	no	no	abnormal	negative	not evaluable	yes
23	23	LAL B	no	no	abnormal	tel-aml1	not evaluable	yes
24	72	LAL B	yes	no	normal	negative	yes	yes
25	39	LAL B	no	no	normal	negative	not evaluable	yes
26	35	LAL B	no	no	abnormal	tel-aml1	no	yes
27	57	LAL B	no	no	abnormal	negative	yes	yes
28	55	LAL B	no	yes	normal	negative	not evaluable	yes
29	149	LAL T	no	no	normal	tel-aml1	yes	yes
30	28	LAL B	no	no	normal	negative	yes	yes
31	51	LAL B	no	no	abnormal	negative	yes	yes
32	23	LAL B	no	no	abnormal	negative	yes	yes
33	97	LAL B	no	no	abnormal	tel-aml1	not evaluable	yes
34	75	LAL B	no	no	normal	negative	yes	yes
35	30	LAL B	no	no	abnormal	mll+	no	yes
36	184	LAL T	no	yes	normal	negative	not evaluable	yes
37	107	LAL B	no	no	normal	negative	yes	yes
38	52	LAL B	no	no	normal	tel-aml1	no	yes
39	43	LAL B	no	yes	abnormal	negative	yes	yes
40	158	LAL B	no	no	normal	negative	yes	yes
41	47	LAL B	no	no	normal	negative	yes	yes
42	58	LAL B	no	no	abnormal	negative	yes	yes
43	147	LAL B	no	no	abnormal	negayive	yes	yes
44	157	LAL B	no	no	normal	negative	yes	yes
45	145	LAL B	no	no	normal	tel-aml1	not evaluable	yes
46	57	LAL B	no	no	abnormal	negative	not evaluable	yes
47	62	LAL B	no	no	abnormal	negative	not evaluable	yes

## DISCUSSION

How maintenance therapy operates in children with ALL remains highly speculative [1, 2]. Several mechanisms of action have been proposed. They include direct long-term cytotoxicity on leukemia cells and inhibition of leukemia stem cell maintenance. The results of the present study provide interesting new insights into how maintenance therapy might affect residual leukemia disease.

In cardiovascular pathologies, the significance of endothelial biomarkers has been clearly established [9]. Indeed, elevated counts of CEC and EMP in the peripheral blood can be considered as markers of endothelial injury and vascular dysfunction, while decrease in EPC, which contribute to endothelium repair processes, plays a critical role in progression of cardiovascular diseases [10]. In clinical oncology, interpretation of these biomarkers levels and kinetics is more complex [11]. CEC and EPC can be considered as surrogate biomarkers of angiogenesis and used for instance to monitor the activity of antiangiogenic agents [12]. The results presented here indicate a significant impact of maintenance therapy on endothelium integrity. The significant decrease in EMP counts and circulating EPC levels underlined a reduction in endothelium activation. Taken together, decreases in these cellular biomarkers and increase in THBS-1 levels, suggest that maintenance therapy reduces endothelium turn-over and restores its resting state. The mechanisms by which maintenance therapy exerts this anti-endothelial effect remains uncertain but several can be proposed. A diresct effect on endothelial cells has been previously demonstrated for the 3 anticancer drugs involved here [3, 13,15]. In addition, this effect could also be mediated by VEGF inhibition and/or THBS-1 up-regulation [3, 7]. In the patients included in this study, VEGF levels remained low and unchanged throughout maintenance therapy; it is thus unlikely that VEGF plays a key role in this setting. In contrast, we observed a significant increase in the level of THBS-1, an antiangiogenic factor and established mediator of MC efficacy [3, 4, 7]. Platelets contain significant amount of bioactive factors such as THBS-1 that can be released during platelet activation and influence neovascularization [14]. It is important to note however that the increase in THBS-1 levels was not associated with significant changes in platelet counts, suggesting that platelets may not be involved in this context. However our study failed to demonstrate a significant inverse correlation between EPCs or EMPs and THBS-1 levels suggesting that THBS-1 may act in concert with other potential mechanisms.

Tregs have recently gained considerable interest from oncologists, as they have been associated with decreased efficacy of treatment in pre-clinical and clinical studies investigating MC [4, 15]. However, no change in Treg numbers was observed as a result of maintenance therapy in our study, suggesting that Tregs are not implicated in the long-term control of ALL. Consistently, Brtnický and al. [16] reported no significant change in Treg numbers in patients with ovarian carcinoma receiving maintenance MC with cyclophosphamide or etoposide. Other immune cell types, such as dendritic cells or myeloid-derived suppressors cells may be affected by maintenance therapy [4, 15]. For instance, Hasnis and al. [17] reported that metronomic gemcitabine improved pancreatic adenocarcinoma outcome by inhibiting the pro-tumorigenic effects induced by myeloid-derived supressor cells. Future studies are required to characterize the evolution of these cell types during ALL maintenance therapy.

The results presented here indicate that maintenance treatment in children with ALL not only pharmacologically but also mechanistically represents a form of metronomic treatment, acting at least in part by decreasing endothelium activity. Larger studies are mandatory to confirm these effects and evaluate their clinical implications. These studies may lead to the identification of novel biomarkers and pave the way to strengthen the antiangiogenic effect of maintenance therapy.

## PATIENTS AND METHODS

### Patients

Details of the population and the underlying leukemia are given in Table 1. Children were all included in the FRALLE protocol and treated accordingly. Maintenance therapy consisted in daily oral purinethol (75 mg/m²/day), weekly oral methotrexate (25 mg/m²/1x/week) and vincristine (1,5 mg/m²) once a month for 12 months and dexamethasone (6 mg/m²/day) for 5 days/month for 12 month. All family and/or patients gave informed consent. The study was approved by ethical and institutional committee. Blood samples were taken before starting maintenance therapy (E0), and respectively after 6 (E6), 12 (E12), and 18 (E18) months of maintenance.

### Enumeration of circulating endothelial cells (CEC)

As previously described [18], CECs were isolated by immuno-magnetic separation using CD146-coated Dynabeads (Life Technologies, Carlsbad, California, USA) from 1mL of whole blood samples collected into EDTA. Isolated cells were then incubated with fluorescein isothiocyanate (FITC) - coupled *Ulex europeaus* lectin-1 (Sigma-Aldrich, St Louis, MO, USA), and quantified with fluorescence microscopy at 533nm. CECs were identified according to the following consensus morphologic and immunologic criteria: rosetted cells with sizes over 15μm in diameter, bearing more than 5 beads and binding *Ulex europeaus* lectin-1. The number of CECs was expressed as cells per mL of blood.

### Enumeration of endothelial microparticles (EMPs)

CD31+ CD41- EMPs were quantified in platelet-poor citrated plasma (PPP). Briefly, PPP was prepared using serial centrifugations (15 min at 1500 × g, 2 min at 13 000 × g) and stored at −80°C until use. Thirty μL of thawed PPP were labeled 30min at room temperature with 10μl of CD41-FITC (Biocytex, Marseille, France), and 10μl of CD31-PE (Beckman Coulter, Miami, FL, USA). 20μl of concentration-matched PE-conjugated murine IgG1 antibody was used as CD31 fluorescence minus one (FMO) control. After incubation, samples were diluted in 500μl of PBS (Life Technologies) and 30μl of flowcount beads (Beckman Coulter) were added for calculation of microparticles absolute values. CD31+ CD41- EMPs were quantified with a FC500 Cytometer (Beckman Coulter) with a previously described standardized flow cytometry protocol for microparticles counting [19]. Results were expressed as EMP per μl of PPP.

### Enumeration of endothelial progenitor cells (EPCs)

Enumeration of CD34+KDR+ EPCs was performed with three-color flow cytometry after direct immunolabeling of peripheral blood mononuclear cells (PBMCs) As previously described [10], PBMCs were isolated from 5 mL of heparinized peripheral blood by density gradient centrifugation with lymphocyte separation medium (PAA Laboratories, Pasching, Austria), and labeled with 10 μL of 7-AAD Viability Dye, 10 μL of FITC–CD34 antibody (Beckman Coulter), and 10 μL of PE–KDR antibody (R&D Systems, Abingdon, UK). 10μl of concentration-matched PE-conjugated murine IgG1 antibody was used as fluorescence minus one (FMO) control. After incubation for 30 min at room temperature, cells were washed, resuspended in 500 μL of phosphate-buffered saline (PBS) (Life Technologies), and analyzed with a FC 500 Cytometer. After selection of 7-AAD-negative cells, KDR+ cells were identified within CD34+ cells displaying FSC/SSC characteristics corresponding to the lymphocyte cluster. At least 5 × 10^5^ viable cells were acquired per run. The percentage of KDR+ cells among CD34+ cells was determined, and absolute values were calculated by multiplying the percentage of KDR+ cells by the absolute values of CD34+ cells. Absolute values of circulating CD34+ cells were determined in whole blood using the single plateform sequential gating strategy according to the standardized ISHAGE protocol [20].

### Enumeration of regulatory T lymphocytes (Tregs)

CD4+ CD25+ Tregs were quantified with a flow cytometry method from whole blood samples collected into EDTA [21]. One hundred μl of samples were labeled 20min at room temperature with 10 μl PE-CD25, Phycoerythrin-Texas Red (ECD)-CD4, and Phycoerythrin-Cyanin5 (PC5)-CD3 antibodies (Beckman Coulter). After lysis of Erythrocytes, cells were washed, resuspended in 500μl of PBS, and analyzed with a FC500 Cytometer. The proportion of CD4+ CD25+ Tregs among CD3+ lymphocytes was determined. Absolute values of Tregs in element mm^−3^ were calculated using the proportion of CD3+ cells obtained by flow cytometry and the lymphocytes numeration obtained from Hemocytometer Instrument.

### Analysis of angiogenic cytokines

Circulating levels of VEGF, VEGF-R1, Thrombospondin-1 (THBS-1), and angiopoietin-2 (ANG-2) were measured in serum using commercially available ELISA kits from R&D System Inc. (Minneapolis, MN, USA). Assays were performed according to manufacturer's instructions.

### Statistical analyses

Statistical analyses were performed using GraphPad Prism Software v.4.0. (GraphPad Software, San Diego, CA, USA). Wilcoxon matched paired test was used to compare different times of maintenance therapy. Differences were considered significant when *p* was < 0.05. To determine the correlation between biomarkers, Spearman correlation test was used with a confidence interval of 95%.
